# Supplementary material for: MetaRibo-Seq measures translation in microbiomes
Source: Nat Commun. 2020 Jun 29;11:3268. doi: 10.1038/s41467-020-17081-z (PMC7324362; doi:10.1038/s41467-020-17081-z)
Supplement: Supplementary file 10 — Supplementary Data 7 [file 41467_2020_17081_MOESM10_ESM.zip › File2/Confidence_High_Taxonomy/133208_out.krona.html]

Javascript must be enabled to view this page.

members
magnitude
magnitudeUnassigned
count
unassigned
taxon
rank

133208\_out

21

21
2
superkingdom

21
976
phylum

class
21
200643

171549
21
order

family
171550
21

239759
21
genus


SRS012969\_contig\_number\_38860SRS013521\_contig\_number\_contig-100\_55.92519SRS016954\_contig\_number\_23505SRS018541\_contig\_number\_13091SRS018623\_contig\_number\_14279SRS020622\_contig\_number\_1714SRS023526\_contig\_number\_contig-100\_1371.222218SRS024625\_contig\_number\_18066SRS043768\_contig\_number\_32500SRS049995\_contig\_number\_19490SRS052576\_contig\_number\_contig-100\_74.75SRS057480\_contig\_number\_10888SRS077849\_contig\_number\_contig-100\_1777.182368SRS078176\_contig\_number\_contig-100\_97.138158SRS098620\_contig\_number\_5239SRS1041118\_contig\_number\_contig-100\_35.100304SRS1054691\_contig\_number\_contig-100\_1317.112446SRS144362\_contig\_number\_42369SRS893187\_contig\_number\_13906SRS893380\_contig\_number\_2766
1872444
20
species

species
28117
1

SRS098655\_contig\_number\_2391
